# Protocol for a caregiver psychosocial support intervention for populations affected by displacement in Uganda

**DOI:** 10.1186/s12889-021-10921-7

**Published:** 2021-05-17

**Authors:** Flora Cohen, Sabrina Hermosilla, Justin Knox, Gary Samuel Agaba, Grace Obalim, Rehema Kajungu, Patrick Onyango Mangen, Lindsay Stark

**Affiliations:** 1 Washington University in St. Louis, Box 1196, 1 Brookings Drive, St. Louis, MO 63130 USA; 2 University of Michigan, 500 S. State St, Ann Arbor, MI 48109 USA; 3 Columbia University, New York, NY 10027 USA; 4 Transcultural Psychosocial Organization Uganda, P.O. Box 21646, Kampala, Uganda; 5 Regional Psychosocial Support Initiative, P.O. Box 23076, Randburg West, South Africa

**Keywords:** Refugee, Psychosocial support, Child protection, Uganda, South Sudan

## Abstract

**Background:**

Child psychological distress in refugee settings is a significant public health concern, which is exacerbated by poor caregiver mental health and functioning. However, there are limited studies about effective interventions to improve caregiver mental health in support of child wellbeing. The objective of the current study is to evaluate the effectiveness and implementation of the Journey of Life (JoL) intervention to improve caregiver mental health in a refugee camp in Western Uganda.

**Methods:**

A waitlist-control quasi-experimental design is being implemented in the Kiryandongo refugee settlement (intervention *n* = 600, control *n* = 600). Caregiver mental distress, measured using the Kessler-6, was selected as the primary outcome. Secondary outcomes include (a) functioning measured by the World Health Organization Disability Assessment Schedule, (b) social support measured by the Medical Outcomes Study Social Support Survey, and (c) caregiving behaviors according to the Parental Acceptance and Rejection Questionnaire and the Child Protection Index. The study aims to examine the implementation of the JoL intervention through qualitative assessments of intervention feasibility, adaptations, and reach.

**Discussion:**

This trial will add much-needed evidence for the implementation of caregiver psychosocial programming within the humanitarian community. Findings will be disseminated amongst local, regional, and global actors in order to guide potential scale up within humanitarian settings.

**Trial registration:**

Clinical Trials NCT04817098 (Registered: 3/24/21).

## Background

Uganda hosts 1.4 million refugees, approximately two thirds of whom are from South Sudan, and over half of whom are children [1]. Displaced children are vulnerable to the impacts of caregiver wellbeing and parenting practices. Refugee caregivers (including biological parents and legal/custodial guardians) cope with a range of stressors including pre-migration conflict and loss as well as post-migration stressors such as poverty, inadequate or unsafe housing, restrictions on employment, limited access to healthcare and education, and loss of social support networks [2–4]. It is well documented that chronic stress leads to negative coping mechanisms such as alcohol and drug abuse, gender based violence, self-harm behaviors, school absenteeism, and worsening mental disorders among adults [5]. Furthermore, caregiver depression is correlated with increased symptoms of depression among their children [6]. High stress also depletes the ability of caregivers to cope and support their children, leading to compromised caregiving, including unresponsive, overprotective, and harsh caregiving [7]. Living in refugee camp settings can strain caregiver wellbeing and parenting behaviors, which in turn can create barriers to healthy child development.

For children, displacement has been shown to amplify susceptibility to malnutrition, infectious disease, prolonged periods out of school, and vulnerability to transactional sex for income and safety [8, 9]. The stressors experienced by children are further exacerbated when adult support mechanisms are strained [10]. Chronically or highly stressed caregivers are more likely to have children with insecure attachments, which has been shown to pose a risk for subsequent difficulties in children’s interpersonal relationships, self-regulation, and achievement [11]. The relationships between caregiver stress, compromised caregiving, and detriments to child wellbeing have been well documented in studies of diverse refugee communities [6, 12–14].

There is growing evidence that psychosocial interventions supporting caregivers can also improve caregiving abilities. Programming that prioritizes caregiving knowledge and skills have been shown to contribute to improved parenting and child outcomes, such as a warmer parenting style, strengthened relationships, and improved academic achievement for children [15–19]. Additionally, psychosocial support interventions with caregivers have demonstrated success in reducing symptoms of caregiver emotional distress, and have shown improvement in overall caregiver well-being [20–28]. However, approaches that build caregiver capacity and support psychosocial well-being have been underutilized and understudied for their effectiveness in humanitarian settings.

Relatedly, the majority of implementation science studies, which assess the uptake of evidence based practices (EBPs) such as caregiver interventions, are based in high income countries [12]. Building the evidence for the implementation of psychosocial EBPs in humanitarian contexts requires the analysis of ecological, institutional, and interpersonal factors including funding and human resource constraints, security and logistics, and a limited capacity of program staff to rigorously assess factors influencing implementation in addition to overall effectiveness [29, 30]. However, a careful investigation into factors that influence implementation in a humanitarian setting is vital to continued uptake of EBPs, scale-up, and dissemination [31–34].

The primary goal of this study is to evaluate the effectiveness and implementation of the Journey of Life (JoL), an intervention to improve child wellbeing through improved caregiver mental health and psychosocial support (MHPSS), in a refugee settlement in Uganda. Through the evaluation, we will:

1. Evaluate the impact of JoL on MHPSS outcomes, including the mental health (primary outcome) of caregivers, along with changes in caregiver functioning, social support, and parenting behaviors (secondary outcomes); and
2. Investigate the implementation of the JoL intervention in terms of feasibility, reach, and adaptations.

## Methods

### Setting

Uganda is one of the most welcoming countries to refugees in the world, with freedom of movement, the right to employment, education, and health. Despite access to these opportunities, 80% of refugees live below the poverty line [35]. Kiryandongo Settlement, the location for this study, has a population of approximately 313,800 people, of whom 17% are refugees; the rest are Ugandan nationals, including internally displaced persons (IDPs). The large majority (99%) of the refugee population in Kiryandongo have fled conflict in South Sudan, while the rest are from the Democratic Republic of Congo, Sudan, Kenya, Burundi, and Rwanda [36]. Approximately 62% of the Kiryandongo population is under the age of 18. In February 2020 there were 847 children identified as unaccompanied, separated, or currently at risk [36]. These numbers are not exhaustive due to incomplete birth registrations and record keeping. Children and adolescents within Kiryandongo consistently report high levels of distress, with 30 to 50% meeting criteria for anxiety and depression [6, 37]. Additionally, 77% of refugees in Kiryandongo reported that when a family member was in psychological distress they were not able to access psychosocial care [38].

### Intervention design

JoL was developed to raise awareness among adults about the psychosocial needs of vulnerable children [39–41]. It provides an opportunity for individuals impacted by conflict and displacement to examine the ways they support children and families in their communities. This JoL adaption focuses on engaging caregivers in building awareness around child protection and fosters psychosocial support through reflection, dialogue, and action. The series of workshops are divided into twelve sessions that include; psychoeducation, self-care, positive parenting, understanding children’s needs, identifying children who need help, and building on children’s strengths. The manualized protocol for 12 sessions is designed to be implemented by non-specialized humanitarian workers. There is an overall emphasis across the curriculum on creating nurturing and caring communities.

### Study design

**Fig. 1:**
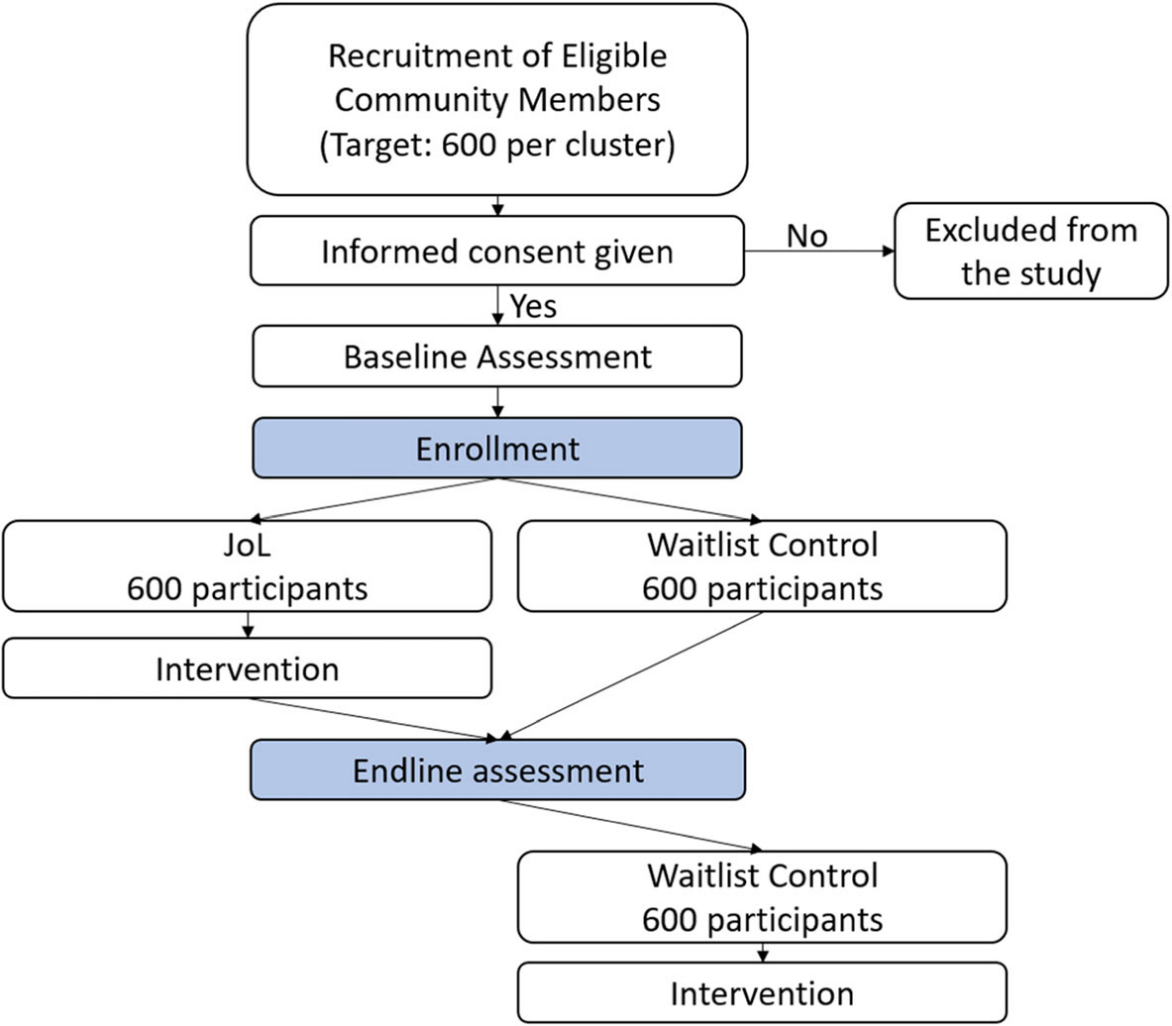
Study flow chart

The proposed study will evaluate the effectiveness and implementation of the JoL intervention using a quasi-experimental waitlist control design. In order to reduce contamination, the treatment group will be conducted in one Ranch (a demarcated segment of the settlement) of Kiryandongo, while the wait-list control group will be in a Ranch that is geographically separate. Participants in the intervention group will receive the program weekly for 12 weeks. The waitlist control group will be invited to participate in the intervention shortly after the endline assessment in the intervention group has been completed (see Fig. 1). Effectiveness indicators will be assessed at baseline and endline.

### Quantitative study design

Every community member (intervention and wait-list control) invited to participate in JoL will also be invited to participate in the evaluation study. To ensure sufficient power, we conducted a sample size calculation for our primary outcome measure – mental distress, measured through the Kessler 6. The proposed sample size (*n* = 960; assuming 80% retention of an initial *n* = 1200) is adequate for detecting an effect size of 11% with 80% power. R (base library, command power.prop.test) was used to conduct the power analysis. We intend to maximize our sample in order to be able to explore potential differences between males and females, and also to be able to look at per-protocol (those who attend > 75% of sessions) versus intent-to-treat analyses.

### Recruitment and population characteristics

Participant recruitment will be conducted in collaboration with the staff of the implementing organization and their community partners. Inclusion criteria for participation in JoL is any person (men and women) ages 18 and over who lives with a child under 18 years old or has caregiving responsibilities for someone under the age of 18. Participants can be refugees or Ugandan nationals living in the study location, they do not need to meet any criteria for adverse mental health, including stress or mild mental illness. Additionally, individuals who have experienced an adverse mental health event are not excluded from the study. Exclusion criteria for the intervention includes anyone aged 17 and under and anyone who is not able to consent to participate.

### Nested qualitative study

Data regarding the implementation of JoL will be collected through qualitative interviews at baseline and endline, and through a process evaluation. Interviewers will conduct key informant interviews (KIIs) with implementing staff, community partners, and participants at baseline and endline. Sample sizes for qualitative data collection will be oriented with the aim of reaching saturation [42]. Data will be analyzed using Dedoose [43]. The process evaluation will draw on routine monitoring tools, including activity sheets, attendance and observation checklists.

### Data management

Participants names will not appear on any collected data; they will each be assigned a unique study ID. Quantitative survey data collected on tablets will be uploaded to the secure server directly by the research coordinator, from which it will be downloaded to Washington University in St. Louis’ secure server and entered into STATA. Qualitative data will be similarly downloaded to a secure server and entered into Dedoose. All non-digital data (e.g. consent forms, activity sheets, and attendance and observation checklists) will be stored securely at the implementation site office, and all data and analysis files will be securely password protected and encrypted. For all quantitative survey data, range and consistently checks will be performed within a few days of data collection. De-identified qualitative data will be translated and transcribed verbatim and entered in Dedoose where they will be coded and analyzed by the research team.

### Adverse events

There is minimal risk to participant safety or wellbeing from participation in this study due to the non-pharmacological and non-clinical nature of this intervention. The occurrence of adverse events (AEs) will be monitored through a standard Adverse Event Reporting Procedure. All AEs reported by enumerators will be recorded by the research team, discussed with the research coordinator and referred to appropriate support services within 24 h of notification.

### Ethics

All study procedures were approved by the Washington University in St. Louis Institutional Review Board (IRB) and by an in-country IRB (TASO Uganda). Eligible participants will be systematically screened by interviewers to determine that they meet the inclusion criteria and are competent to be interviewed. Everyone participating in the intervention will be invited to participate in the baseline and endline surveys to track participant changes. Data collection staff will be trained and available to respond to any questions on the consenting process. Consent will be explained to participants verbally and in a written format, participants will sign written consent for participation.

### Effectiveness outcomes

All questionnaire data will be gathered using Android tablets. Measures will be administered in Juba Arabic, Dinka, Nuer, and Acholi (languages covering the majority of the population) by trained and supervised enumerators. In order to capture key demographic information, the survey contains questions related to age, gender, marital status, years of education, current sources of income, and caregiving status. Survey modules cover functioning, social support, experiences of intimate partner violence, mental distress, child protection behaviors, and parenting styles. See Table 1 for a detailed overview of measures.

**Table 1:** Outcome Measures

Measure	Tool	Description
Mental distress	Kessler-6	The Kessler-6 asks participants how frequently they have experienced certain symptoms in the past thirty days and presents a consistent range of responses ranging from “1 - All of the time” to “5 - None of the time” [44]. The scale shows consistency across multiple socio-demographic variables and has been previously documented among refugees in Uganda [45].
Functioning	World Health Organization Disability Assessment Schedule	WHODAS assesses functioning through six domains of cognition, mobility, self-care, interpersonal skills, life activities, and community participation. The WHODAS has been used in international settings including Uganda Bachani et al. [46].
Social Support	Medical Outcomes Study (MOS)	The MOS measures social support across five dimensions, including: 1) emotional, 2) informational, 3) tangible, 4) positive social interactions, and 5) affection. It has been used to assess social support among individuals affected by HIV in Uganda [47–49].
Parenting behaviors	Parenting Acceptance and Rejection Questionnaire	For the purposes of this study, the subscales of warmth/affection, hostility/aggression, indifference/neglect, and undifferentiated rejection will be used. The PARQ has been used in similar settings to assess parenting behaviors [50].
Attitudes towards child protection	Child Protection Index	Items related to perceptions on the treatment of children, child rearing, and educating children are included in this study. Developed in collaboration with the UNHCR, AVSI, and the CPC Learning Network, the Child Protection Index, assesses child protection outcomes in displacement settings Meyer et al. [51], and has been used previously in Uganda Meyer et al., [6, 52, 53].
Intimate partner violence	WHO multi-country Violence Against Women (VAW) study	These items estimate the prevalence of physical, sexual, and emotional violence against women. The World Health Organization (WHO) Multi-country study on Women’s Health and Domestic Violence included the collection of data from over 24,000 women in 10 countries.

### Implementation measures

Frameworks such as FRAME (Framework for Reporting Adaptations and Modifications-Expanded) provides a structure to analyze program adaptations pre-, during, and post-implementation [54]. FRAME includes [1] when and how adaptations were made, [2] whether the adaptations were unplanned/reactive or planned/proactive, [3] who determined the adaptation, [4] what was adapted, [5] at what level of delivery the adaptation was made, [6] the type of adaptation, [7] the extent to which the adaptation is consistent with fidelity, [8] the reasons for the adaptation. Adaptations, including the successful tailoring of evidence-based practices for the target population, may improve participant involvement and overall clinical outcomes.

Additionally, components of the CFIR (Consolidated Framework for Implementation Research) [55] will be integrated into qualitative semi-structured KIIs at baseline and endline with implementation staff, community organization partners, and community members in order to address implementation components, including acceptability, feasibility, and others outlined in Table 2. Findings from KIIs will be used to inform program adaptations and record implementation indicators. Additional indicators will be tracked using monitoring and evaluation (M&E) approaches such as tracking attendance, and monitoring fidelity to program components using observation checklists.

**Table 2:** Implementation science framework for JoL assessment

Construct	Short Description	Collection Method
**Intervention characteristics**		
Acceptability	Define if the program feels acceptable to community stakeholders	KII
**Outer setting**		
Needs & Resources	Community needs and available supportive resources.	KII
Cosmopolitanism	Organizational network and connections with other organizations.	KII
**Inner setting**		
Culture	Organizational norms, values, and customs.	KII
Feasibility	Defining barriers and facilitators in order to address potential adaptions to the implementation strategy and manualized tools.	KII
**Characteristics of individuals**		
Self-efficacy	Belief in one’s own capabilities to execute the implementation plan.	KII
Attendance	Track attendance at weekly sessions and reasons for not attending	M&E
Execution	Delivering the program according to plan.	KII
**JoL Components**		
Adaptations	Tailoring the intervention to population and context specific needs.	KII
Group composition	The composition of groups, including demographics of participants.	
Space procurement	The proposed location of the JoL groups.	KII
Scheduling	The best time of day and/or day of the week to meet	KII
Attrition	Barriers and facilitators to retention	KII
Fidelity	Track fidelity to program components using observation checklists	M&E

### Data analysis

#### Quantitative analysis

The analytic plan is designed to assess effectiveness of the JoL intervention on MHPSS and caregiving behaviors. The level of significance for all analyses will be set at 5%. We will initially assess the sample in terms of baseline characteristics. Other preliminary data analyses will include studies of patterns of missing data, dropout rates, distributional properties of dependent and other measures, and correlations among outcomes. We will determine if effectiveness differs meaningfully in the intervention group compared to the control group. As the primary intervention is community based, an individual’s specific exposure to intervention activities (e.g. intervention dose received) will not be explored, but rather all individuals within the intervention group will be categorized as intervention respondents and all individuals within the control group will be categorized as control respondents. We will also assess the effect of potential mediators in the causal pathway between treatment and outcome. To explore if efficacy differs by baseline characteristics (moderation), we will model the outcomes with the relevant baseline characteristics and their interaction as predictors. Factor-stratified models (e.g. by gender) may be explored if interaction terms are found to be statistically significant. Our modeling approach to assess changes in outcomes over time will use generalized mixed models for longitudinal data. This class of models does not assume that subjects are measured at all time-points, and therefore can include subjects with two measurement time-points. In these analyses, we will examine changes across time in order to assess – and isolate from programmatic efficacy - the effect of time. Based on intervention attendance records, both intention-to-treat and per-protocol analyses will be explored. We will also conduct sensitivity analyses to investigate the robustness of results across different models for missing data, following this detailed approach. Additional analyses will also be explored, such as the need for models that use inverse probability weighting to account for potentially high levels of incomplete intervention participation within the intervention community.

#### Qualitative analysis

On-going qualitative analyses will be conducted over the course of the study to explore new and unexpected themes that arise; we will shift to new questions when saturation is reached. Using Dedoose, multiple study staff members will independently code 10% of the transcripts and compare the application of the coding scheme to assess its reliability and robustness; we will resolve disagreements through discussion.

Systematic analysis of the various types of data collected will be conducted using a grounded theory approach. Grounded theory analysis requires that data be analyzed continuously throughout the duration of data collection, so that theories generated from the data can be used to direct and inform subsequent research efforts. In grounded theory, the researcher summarizes initial observations into conceptual categories and then tests the coherence of the categories in the research setting with additional observations so that theory evolves but is grounded in data from subsequent observations. An initial codebook and coding scheme will be developed and expanded as the data are analyzed.

We will use the content analysis techniques, *repetitions* and *cutting and sorting*, to identify themes in the data. These content analyses techniques involve identifying the recurrence of words/phrases (*repetitions*), and recognizing patterns in quotes and expressions that are salient and that can be distinguished from other patterns in the data (*cutting*), then organizing them together (*sorting*).

#### Dissemination

The results of this project will be disseminated internally among implementation staf of TPO and REPSSI about the use and implementation of JoL. Results will be shared in verbal and written formats to ensure accessibility. Additionally, presentations about outcomes will be organized for UNHCR agency meetings both in settlement locations and among agencies in Kampala. Other forums for dissemination will include annual research meetings and forums virtually and in-person. If JoL is proven to be effective, the adapted manual will be shared with program developers, implementation staff, and key partners in order to facilitate scale up of implementation. REPSSI will also disseminate the adapted manual across countries of operation. Furthermore, results will be published in English in peer-reviewed journals for regional and global audiences.

## Discussion

Although psychosocial programs comprise a large proportion of MHPSS humanitarian programming for mental health and wellbeing, evidence to support the effectiveness of psychosocial interventions remains nascent [56]. Psychosocial programming remains conceptually different from the treatment of mental disorders through mental health programming, and includes a broader array of potential intervention approaches. This breadth presents a challenge in the field, as boundaries of what is meant by ‘psychosocial wellbeing’ remain nebulous. For example, a series of impact evaluations recently found that widely-implemented child-friendly spaces (CFS), which have generally been perceived to be instrumental for psychosocial wellbeing and child protection, have small to no effects (potentially related to quality and fit to local context) [57–59]. One of the lessons learned from this body of research is that the provision of space alone is not sufficient to improve psychosocial wellbeing, but programming that supports social support and psychological wellbeing alongside the provision of basic services is vital.

Even when there is sufficient evidence for programs, they are rarely scaled or widely implemented [60]. In recent years, there has been a shift in research to ensure that evidence-informed programs, such as JoL, incorporate rigorous tracking of implementation strategies to strengthen future scalability, replicability, and sustainability. Ideally, psychosocial programs can continue to be implemented and have an impact on communities long after the program is first initiated. Implementation strategies, guided by implementing organizations and iteratively improved, help to maximize the effectiveness of programs within each community, while being mindful of contextual factors and available resources. This study tests the effectiveness of a seldom-studied psychosocial intervention, while simultaneously gathering information about its implementation in a real world setting [61]. This hybrid design is particularly important in areas with limited resources, where factors to improve the implementation and sustainability of programs are lesser known and where there are barriers to rigorous implementation trials [61–65].

We acknowledge potential challenges that may emerge during the implementation and evaluation of the JoL program. One of these challenges includes operating during the COVID-19 pandemic. Good practice safety measures have been put in place to protect the health of staff and participants involved in the program and evaluation. Another challenge may involve operating during the wet season in Uganda, which may affect access and participation and will be monitored as part of program M&E. Additionally, we have tried to account for potential spillover effects by ensuring the selected Ranches are sufficiently geographically separated. We know that groups of participants will include community members who hold various roles in the community who may engage with program material differently; we will be carefully monitoring and working to minimize power dynamics within the groups. Implementation and evaluation staff will monitor emerging issues, and provide a constructive environment for participation.

The primary goal of JoL is to provide a sustainable solution to support the wellbeing of children and families living in Kiryandongo, Uganda. Following intervention delivery, it is hypothesized that participants will have improved self-care strategies and be better able to promote and improve child wellbeing. Overall, this study supports burgeoning literature about caregiver psychosocial support and child protection programs in humanitarian and low-resourced settings.

## Data Availability

Not Applicable.

## Abbreviations

JoL: Journey of Life
EBP: Evidence Based Practice
MHPSS: Mental Health and Psychosocial Support
AE: Adverse Events
IRB: Institutional Review Board
WHODAS: World Health Organization Disability Assessment Schedule
MOS: Medical Outcomes Study
PARQ: Parenting Acceptance and Rejection Questionnaire
UNHCR: United Nations High Commissioner for Refugees
ASVI: Association of Volunteers in International Service
CPC: Child Protection in Crisis
VAW: Violence Against Women
FRAME: Framework for Reporting Adaptations and Modifications-Expanded
CFIR: Consolidated Framework Implementation Research
KII: Key Informant Interview
M&E: Monitoring and Evaluation
TPO: Transcultural Psychosocial Organization
REPSSI: Regional Psychosocial Support Initiative
CFS: Child-Friendly Spaces

## References

[1] UNICEF (2021). UNICEF Uganda’s emergency response to refugees.

[2] Kibret B (2015). Armed conflict, violation of child rights and implications for change. J Psychol Psychother.

[3] MacMullin C, Loughry M (2004). Investigating psychosocial adjustment of former child soldiers in Sierra Leone and Uganda. J Refug Stud.

[4] Paardekooper B, de Jong JT, Hermanns JM (1999). The psychological impact of war and the refugee situation on south Sudanese children in refugee camps in northern Uganda: an exploratory study. J Child Psychol Psychiatry.

[5] Kaltenbacher S (2019). UNHCR Uganda mental health and psychosocial support strategy.

[6] Meyer SR, Steinhaus M, Bangirana C, Onyango-Mangen P, Stark L (2017). The influence of caregiver depression on adolescent mental health outcomes: findings from refugee settlements in Uganda. BMC Psychiatry.

[7] Biglan A, Flay BR, Embry DD, Sandler IN (2012). The critical role of nurturing environments for promoting human well-being. Am Psychol.

[8] UNESCO (2016). Impact of conflict on children’s health and disability.

[9] Schlecht J, Rowley E, Babirye J (2013). Early relationships and marriage in conflict and post-conflict settings: vulnerability of youth in Uganda. Reprod Health Matters.

[10] Reed RV, Fazel M, Jones L, Panter-Brick C, Stein A (2012). Mental health of displaced and refugee children resettled in low-income and middle-income countries: risk and protective factors. Lancet.

[11] Miller KE, Arnous M, Tossyeh F, Chen A, Bakolis I, Koppenol-Gonzalez GV, Nahas N, Jordans MJD (2020). Protocol for a randomized control trial of the caregiver support intervention with Syrian refugees in Lebanon. Trials..

[12] El-Khani A, Maalouf W, Baker D, Zahra N, Noubani A, Cartwright K (2020). Caregiving for children through conflict and displacement: a pilot study testing the feasibility of delivering and evaluating a light touch parenting intervention for caregivers in the West Bank. Int J Psychol.

[13] Puffer ES, Green EP, Chase RM, Sim AL, Zayzay J, Friis E, Garcia-Rolland E, Boone L (2015). Parents make the difference: a randomized-controlled trial of a parenting intervention in Liberia. Glob Ment Health Camb Engl.

[14] Sim A, Annan J, Puffer E, Salhi C, Betancourt T. Building happy families: impact evaluation of a parenting and family skills intervention for migrant and displaced Burmese families in Thailand. N Y Int Rescue Comm. 2014. Available from: https://www.researchgate.net/publication/306268893_Building_Happy_Families_Impact_evaluation_of_a_parenting_and_family_skills_intervention_for_migrant_and_displaced_Burmese_families_in_Thailand

[15] Bass J, Annan J, Murray S, Kaysen D, Griffiths S, Cetinoglu T (2013). Controlled trial of psychotherapy for Congolese survivors of sexual violence. NEJM..

[16] Bunn M, Goesel C, Kinet M, Ray F (2015). Group therapy with male asylum seekers and refugees with posttraumatic stress disorder: a controlled comparison cohort study of three day-treatment programs. Torture..

[17] Falb KL, Tanner S, Ward L, Erksine D, Noble E, Assazenew A (2016). Creating opportunities through mentorship, parental involvement, and safe spaces (COMPASS) program: multi-country study protocol to protect girls from violence in humanitarian settings. BMC Public Health.

[18] Falb KL, Asghar K, Laird B, Tanner S, Graybill E, Mallinga P, Stark L (2017). Caregiver parenting and gender attitudes: associations with violence against adolescent girls in south Kivu, Democratic Republic of Congo. Child Abuse Negl.

[19] Stark L, Seff I, Asghar K, Roth D, Bakamore T, MacRae M, Fanton D’Andon C, Falb KL (2018). Building caregivers’ emotional, parental and social support skills to prevent violence against adolescent girls: findings from a cluster randomised controlled trial in Democratic Republic of Congo. BMJ Glob Health.

[20] Goodkind JR, Hess JM, Isakson B, LaNoue M, Githinji A, Roche N, Vadnais K, Parker DP (2014). Reducing refugee mental health disparities: a community-based intervention to address postmigration stressors with African adults. Psychol Serv.

[21] Kinzie J, Kinzie J, Sedighi B, Woticha A, Mohamed H, Riley C (2012). Prospective one-year treatment outcomes of tortured refugees: a psychiatric approach. Torture.

[22] Lambert J, Alhassoon O (2015). Trauma-focused therapy for refugees: meta-analytic findings. J Couns Psychol.

[23] Nosè M, Ballette F, Bighelli I, Turrini G, Purgato M, Tol W, Priebe S, Barbui C (2017). Psychosocial interventions for post-traumatic stress disorder in refugees and asylum seekers resettled in high-income countries: systematic review and meta-analysis. PLoS One.

[24] Shaw SA, Ward KP, Pillai V, Hinton DE (2019). A group mental health randomized controlled trial for female refugees in Malaysia. Am J Orthop.

[25] Tol WA, Barbui C, Galappatti A, Silove D, Betancourt TS, Souza R, Golaz A, van Ommeren M (2011). Mental health and psychosocial support in humanitarian settings: linking practice and research. Lancet Lond Engl.

[26] Palic S, Elklit A (2011). Psychosocial treatment of posttraumatic stress disorder in adult refugees: a systematic review of prospective treatment outcome studies and a critique. J Affect Disord.

[27] Slobodin O, de Jong JTVM (2015). Mental health interventions for traumatized asylum seekers and refugees: what do we know about their efficacy?. Int J Soc Psychiatry.

[28] Weiss WM, Ugueto AM, Mahmooth Z, Murray LK, Hall BJ, Nadison M (2016). Mental health interventions and priorities for research for adult survivors of torture and systematic violence: a review of the literature. Torture Q J Rehabil Torture Vict Prev Torture.

[29] Ridde V (2016). Need for more and better implementation science in global health. BMJ Glob Health.

[30] Dickson K, Bandpan M (2018). What are the barriers to, and facilitators of, implementing and receiving MHPSS programmes delivered to populations affected by humanitarian emergencies? A qualitative evidence synthesis. Glob Ment Health.

[31] Powell BJ, Beidas RS, Lewis CC, Aarons GA, McMillen JC, Proctor EK (2017). Methods to improve the selection and tailoring of implementation strategies. J Behav Health Serv Res.

[32] Cohen F, Yaeger L (2021). Task-shifting for refugee mental health and psychosocial support: a scoping review of services in humanitarian settings through the lens of RE-AIM. Implement Res Pract.

[33] Perera C, Salamanca-Sanabria A, Caballero-Bernal J, Feldman L, Hansen M, Bird M (2020). No implementation without cultural adaptation: a process for culturally adapting low-intensity psychological interventions in humanitarian settings. Confl Health.

[34] Tol WA, Augustinavicius J, Carswell K, Leku MR, Adaku A, Brown FL, García-Moreno C, Ventevogel P, White RG, Kogan CS, Bryant R, van Ommeren M (2018). Feasibility of a guided self-help intervention to reduce psychological distress in south Sudanese refugee women in Uganda. World Psychiatry.

[35] UNHCR (2021). COVID-19 Supplementary Appeal 2021.

[36] UNHCR (2020). Uganda Active Population by Settlement December 2020.

[37] Meyer SR, Yu G, Rieders E, Stark L (2020). Child labor, sex and mental health outcomes amongst adolescent refugees. J Adolesc.

[38] UNHCR. Joint Multi-Sector Needs Assessment - Identifying humanitarian needs among refugee and host community populations in Uganda (August 2018) - Uganda. ReliefWeb. 2018 [cited 2021 Jan 25]. Available from: https://reliefweb.int/report/uganda/joint-multi-sector-needs-assessment-identifying-humanitarian-needs-among-refugee-and

[39] Lanhuang L, Adefrsew A (2013). An evaluation of journey of life implementation within pact Yekokeb Berhan program.

[40] Matikanya R, James V, Maksud N (2006). End of programme support evaluation of regional psychosocial support initiative (REPSSI).

[41] The Government of Malawi (2016). Journey of life: community awareness and mobilisation tool in achieving child protection results in Malawi.

[42] Vasileiou K, Barnett J, Thorpe S, Young T. Characterising and justifying sample size sufficiency in interview-based studies: systematic analysis of qualitative health research over a 15-year period. BMC Med Res Methodol. 2018; 21 [cited 2021 Mar 23];18. Available from: https://www.ncbi.nlm.nih.gov/pmc/articles/PMC6249736/. 10.1186/s12874-018-0594-7 PMC6249736 30463515

[43] Dedoose. Los Angeles, CA: SocioCultural Research Consultants, LLC; 2018. Available from: www.dedoose.com

[44] Kessler RC, Barker PR, Colpe LJ, Epstein JF, Gfroerer JC, Hiripi E, Howes MJ, Normand S-L T, Manderscheid RW, Walters EE, Zaslavsky AM. Screening for Serious Mental Illness in the General Population. Arch Gen Psychiatry. 2003;60(2):184. 10.1001/archpsyc.60.2.184 12578436

[45] Tol WA, Leku MR, Lakin DP, Carswell K, Augustinavicius J, Adaku A, Au TM, Brown FL, Bryant RA, Garcia-Moreno C, Musci RJ, Ventevogel P, White RG, van Ommeren M (2020). Guided self-help to reduce psychological distress in south Sudanese female refugees in Uganda: a cluster randomised trial. Lancet Glob Health.

[46] Bachani AM, Galiwango E, Kadobera D, Bentley JA, Bishai D, Wegener S, Zia N, Hyder AA (2016). Characterizing disability at the Iganga-Mayuge demographic surveillance system (IM-DSS), Uganda. Disabil Rehabil.

[47] Bajunirwe F, Tisch DJ, King CH, Arts EJ, Debanne SM, Sethi AK (2009). Quality of life and social support among patients receiving antiretroviral therapy in Western Uganda. AIDS Care.

[48] Stangl AL, Bunnell R, Wamai N, Masaba H, Mermin J (2012). Measuring quality of life in rural Uganda: reliability and validity of summary scores from the medical outcomes study HIV health survey (MOS-HIV). Qual Life Res.

[49] Takada S, Weiser SD, Kumbakumba E, Muzoora C, Martin JN, Hunt PW, Haberer JE, Kawuma A, Bangsberg DR, Tsai AC (2014). The dynamic relationship between social support and HIV-related stigma in rural Uganda. Ann Behav Med.

[50] Stark L, Asghar K, Seff I, Yu G, Tesfay Gessesse T, Ward L, Assazenew Baysa A, Neiman A, Falb KL (2018). Preventing violence against refugee adolescent girls: findings from a cluster randomised controlled trial in Ethiopia. BMJ Glob Health.

[51] Meyer DS, Steinhaus M, Stark DL (2015). Measuring impact through a child protection index.

[52] Measuring Impact Through a Child Protection Index (2014). CPC Learning Network.

[53] Meyer SR, Yu G, Hermosilla S, Stark L. Latent class analysis of violence against adolescents and psychosocial outcomes in refugee settings in Uganda and Rwanda. Glob Ment Health. 2017; [cited 2020 Apr 28];4. Available from: https://www.cambridge.org/core/journals/global-mental-health/article/latent-class-analysis-of-violence-against-adolescents-and-psychosocial-outcomes-in-refugee-settings-in-uganda-and-rwanda/9A2BB75FBF331D5C3D7024FB36317DFC. 10.1017/gmh.2017.17 PMC5719474 29230315

[54] Wiltsey Stirman S, Baumann AA, Miller CJ (2019). The FRAME: an expanded framework for reporting adaptations and modifications to evidence-based interventions. Implement Sci.

[55] Keith RE, Crosson JC, O’Malley AS, Cromp D, Taylor EF (2017). Using the consolidated framework for implementation research (CFIR) to produce actionable findings: a rapid-cycle evaluation approach to improving implementation. Implement Sci IS.

[56] Lee C, Nguyen AJ, Haroz E, Tol W, Aules Y, Bolton P. Identifying research priorities for psychosocial support programs in humanitarian settings. Glob Ment Health. 2019; 7 [cited 2021 Mar 24];6. Available from: https://www.ncbi.nlm.nih.gov/pmc/articles/PMC6796323/. 10.1017/gmh.2019.19 PMC6796323 31662878

[57] Ager A, Metzler J, Vojta M, Savage K (2013). Child friendly spaces: a systematic review of the current evidence base on outcomes and impact. Intervention..

[58] Hermosilla S, Metzler J, Savage K, Musa M, Ager A (2019). Child friendly spaces impact across five humanitarian settings: a meta-analysis. BMC Public Health.

[59] Metzler J, Savage K, Yamano M, Ager A (2015). Evaluation of child friendly spaces: an inter-agency series of impact evaluations in humanitarian emergencies [internet].

[60] Morris Z, Wooding S, Grant J (2011). The answer is 17 years, what is the question: understanding time lags in translational research. J Roy Soc Med.

[61] Curran GM, Bauer M, Mittman B, Pyne JM, Stetler C (2012). Effectiveness-implementation hybrid designs. Med Care.

[62] Bernet AC, Willens DE, Bauer MS (2013). Effectiveness-implementation hybrid designs: implications for quality improvement science. Implement Sci.

[63] Damschroder LJ, Moin T, Datta SK, Reardon CM, Steinle N, Weinreb J, Billington CJ, Maciejewski ML, Yancy WS, Hughes M, Makki F, Richardson CR (2015). Implementation and evaluation of the VA DPP clinical demonstration: protocol for a multi-site non-randomized hybrid effectiveness-implementation type III trial. Implement Sci.

[64] Ma J, Yank V, Lv N, Goldhaber-Fiebert JD, Lewis MA, Kramer MK, Snowden MB, Rosas LG, Xiao L, Blonstein AC (2015). Research aimed at improving both mood and weight (RAINBOW) in primary care: a type 1 hybrid design randomized controlled trial. Contemp Clin Trials.

[65] Smith JD, Stormshak EA, Kavanagh K (2015). Results of a pragmatic effectiveness–implementation hybrid trial of the family check-up in community mental health agencies. Admin Pol Ment Health.

